# Supplementary material for: Causes and consequences of sex-chromosome turnovers in Diptera
Source: Evol Lett. 2026 Mar 12;10(3):253–68. doi: 10.1093/evlett/qrag003 (PMC13231274; doi:10.1093/evlett/qrag003)
Supplement: qrag003_Supplemental_Files [file qrag003_supplemental_files.zip › Supplementary_Datasets.zip!/Supplementary_Datasets/Dataset_2_Graphical_representation_outgroups/Syrphidae_Eristalis_tenax_chromosomes.pdf]

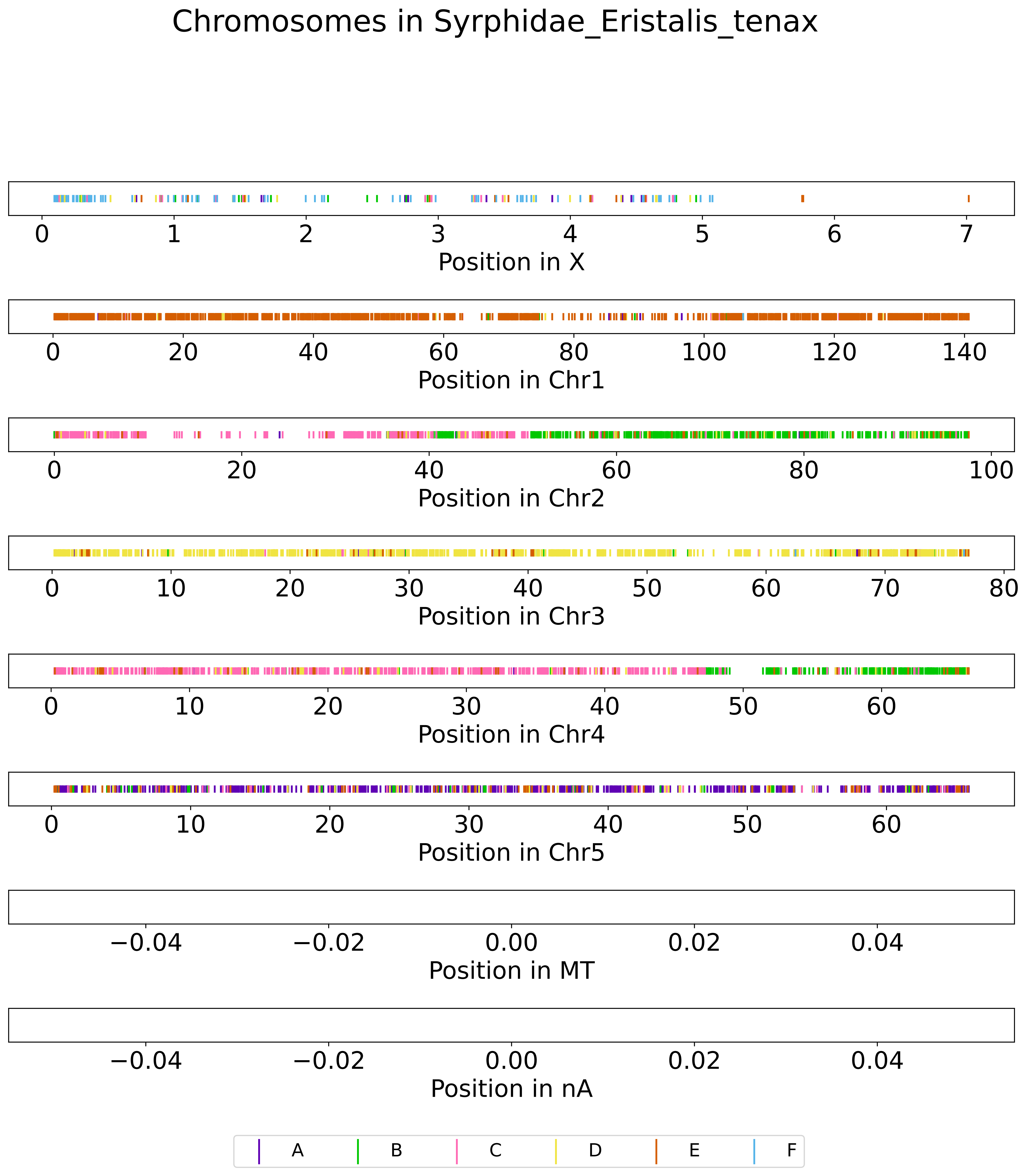

Chromosomes in Syrphidae_Eristalis_tenax
0 1 2 3 4 5 6 7
Position in X
0 20 40 60 80 100 120 140
Position in Chr1
0 20 40 60 80 100
Position in Chr2
0 10 20 30 40 50 60 70 80
Position in Chr3
0 10 20 30 40 50 60
Position in Chr4
0 10 20 30 40 50 60
Position in Chr5
−0.04 −0.02 0.00 0.02 0.04
Position in MT
−0.04 −0.02 0.00 0.02 0.04
Position in nA
A
B
C
D
E
F
